# Clamp On vs Off Impact of Distal Anastomotic Technique During Ascending Aortic Replacement in Acute Type A Aortic Dissection: IRAD Insights

**DOI:** 10.1093/icvts/ivag093

**Published:** 2026-04-03

**Authors:** Stevan S Pupovac, Omar A Jarral, Santi Trimarchi, Ibrahim Sultan, Bradley Leshnower, Nimesh D Desai, Thoralf M Sundt, Puja Kachroo, Qing-Guo Li, Chih-Wen Pai, Thomas G Gleason, Himanshu J Patel, Kim A Eagle, Derek R Brinster

**Affiliations:** Department of Cardiovascular & Thoracic Surgery, Northwell Health, Manhasset, NY, 11103, USA; Department of Cardiovascular & Thoracic Surgery, Northwell Health, Manhasset, NY, 11103, USA; Department of Scienze Cliniche e di Comunita/Fondazione IRCCS Ca’ Granda Ospedale Maggiore Policlinico, University of Milan, Milan, 20122, Italy; Department of Cardiothoracic Surgery, University of Pittsburgh, Pittsburgh, PA, 15213, USA; Division of Cardiothoracic Surgery, Emory University, Atlanta, GA, 30322, USA; Department of Cardiovascular Surgery, University of Pennsylvania School of Medicine, Philadelphia, PA, 19104, USA; Department of Surgery, Massachusetts General Hospital, Boston, MA, 02114, USA; Cardiovascular Surgery, Washington University School of Medicine, St. Louis, MO, 63110, USA; Department of Cardiovascular Surgery, Nanjing Medical University, Nanjing, 210029, China; Frankel Cardiovascular Center, University of Michigan, Ann Arbor, MI, 48109, USA; Cardiovascular Surgery, Asheville Heart, Asheville, NC, 28801, USA; Frankel Cardiovascular Center, University of Michigan, Ann Arbor, MI, 48109, USA; Frankel Cardiovascular Center, University of Michigan, Ann Arbor, MI, 48109, USA; Department of Cardiovascular & Thoracic Surgery, Northwell Health, Manhasset, NY, 11103, USA

**Keywords:** aorta, aortic dissection, circulatory arrest

## Abstract

**Objectives:**

Most ascending aortic replacements for acute type A aortic dissection (ATAAD) are performed utilizing hypothermic circulatory arrest with an “open” distal anastomosis (“hemiarch”), whereas some are completed without ever removing the aortic cross-clamp. We sought to determine the impact of distal anastomotic technique in ascending aortic replacement for ATAAD repair.

**Methods:**

All patients in the IRAD Interventional Cohort database who underwent ATAAD repair between 2010 and 2020 were identified (*n* = 2559). Data for distal anastomotic technique were available in 2031 patients who underwent ascending aortic replacement, divided into 2 groups, based on whether they underwent hypothermic circulatory arrest (Clamp Off, *n* = 1900 [93.5%]) or not (Clamp On, *n* = 131, [6.5%]). We then propensity-matched 101 pairs of patients and analysed operative data and short- and mid-term outcomes.

**Results:**

In-hospital mortality for the unmatched population was 13.1% (266 deaths), not statistically different between the matched groups (Clamp Off, 11.0% vs Clamp On, 5.1% *P* = .22). There were no statistically significant differences observed in 3-year post-discharge survival curves between matched cohorts (Clamp Off, 89.5% vs Clamp On, 90.4%; stratified log-rank *P-*value = .46). Major perioperative complications (renal failure requiring dialysis, reoperation for bleeding, respiratory insufficiency) were not significantly different between the groups, notably including stroke (Clamp Off, 10.2% vs Clamp On, 5.8%, *P* = .32).

**Conclusions:**

In this propensity-matched cohort, postoperative mortality, major morbidity, and mid-term survival were comparable between open distal anastomosis and clamp-on strategies in selected patients undergoing ascending aortic replacement for ATAAD.

## Graphical abstract

**Figure:**
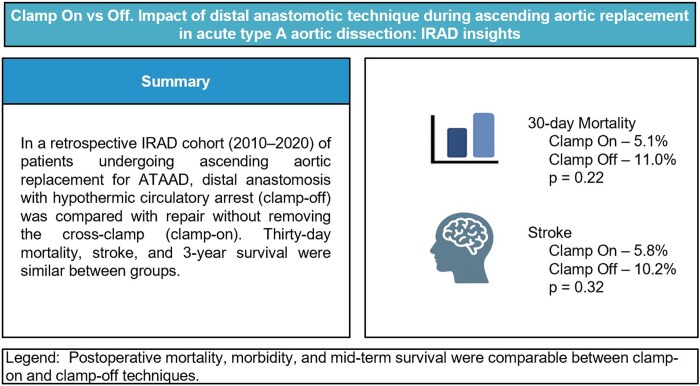

## INTRODUCTION

Despite advances in surgical technique and perioperative care, acute Stanford type A aortic dissection (ATAAD) remains associated with significant mortality, typically quoted upwards of 18%.^1–3^ Recent data from the International Registry of Acute Aortic Dissection (IRAD) report an overall surgical mortality of roughly 12%, an improvement over the past 2 decades, but substantial nonetheless.^2^

While considerable attention has been devoted to improvements in ATAAD repair, such as adjunctive cerebral perfusion techniques,^4–6^ varying levels of hypothermic circulatory arrest (HCA),^7–11^ and hemostasis,^12^ relatively little focus has been placed on evaluating the impact of distal anastomotic technique—a fact that was noticed by Myrmel et al over 2 decades ago.^13^

Standard treatment of ATAAD involves replacement of the diseased ascending aorta along with concomitant resection of the intimal entry tear. Currently, there is consensus that the distal anastomosis should be performed using an “open” distal anastomotic technique (“hemiarch”) under varying levels of HCA as opposed to maintaining the aortic cross-clamp on for the duration of the repair (“clamp on”).^14^^,^^15^ Although the Society of Thoracic Surgeons (STS) and European Association for Cardio-Thoracic Surgery (EACTS) guidelines give a Class IB recommendation for an open distal anastomosis, the supporting data are limited and not entirely consistent. The STS and EACTS guidelines cite only 2 sources for this recommendation,^16^^,^^17^ while most published studies are equivocal regarding operative mortality and short- to mid-term survival.^18–21^

With this in mind, we sought to evaluate the outcomes of ATAAD repair, comparing those patients who underwent surgery utilizing an “open” distal anastomosis, compared to those who had their repair without ever removing the aortic cross-clamp.

## METHODS

### Study population

This analysis included all ATAAD patients enrolled in the IRAD Interventional Cohort (IRAD-IVC) from 2010 to 2020. IRAD captures data from 72 large referral centres across 16 countries, with Institutional Review Board approval obtained at each participating site. Given the retrospective, observational design of the IRAD registry and the use of de-identified data from multiple international institutions, the requirement for individual informed consent was waived by the institutional review board or ethics committee at each participating site, in accordance with local regulations.

**Figure 1.**
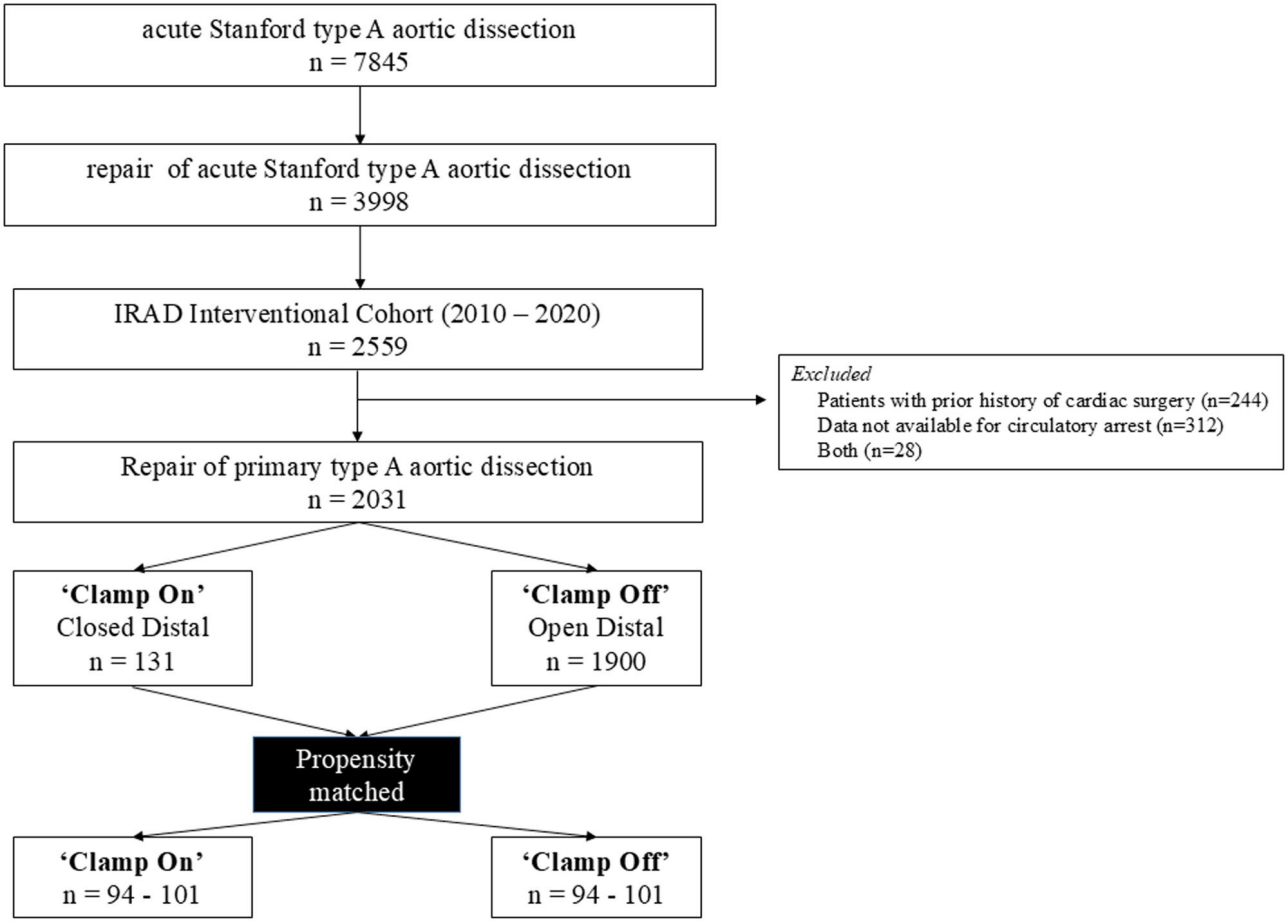
Flow Chart of Patient Selection

ATAAD was defined as any dissection involving the ascending aorta presenting within 14 days of symptom onset. Patients were excluded if definitive circulatory arrest data were unavailable or if they had a history of prior cardiac surgery. Patients were then classified according to whether repair was performed with an open distal anastomosis or without removing the aortic cross-clamp (**Figure 1**).

### Data collation and definitions

Data were submitted by participating institutions to the IRAD Coordinating Center at the University of Michigan. Definitions of demographic, anatomic, perioperative, and postoperative variables were derived from the IRAD Acute Form Lexicon v7.0, Follow-up Form Lexicon v7.0, and Invasive Treatment Data Form Definitions v6.1.

Dissection extent was defined using IRAD registry variables capturing the most proximal and distal extent of the intimal flap (ascending aorta, arch, descending thoracic aorta, or beyond). Although ATAAD required involvement of the ascending aorta, IRAD coding permits rare categorization of proximal extent at the level of the arch; these cases were included because they were managed surgically as ATAAD. A prespecified sensitivity analysis excluding such cases was performed to confirm robustness of the findings. Image-derived anatomic and operative variables were not included in multiple imputation; patients with missing key matching variables (arterial cannulation site, aortic root replacement, and distal dissection extent) were excluded from the propensity score model.

### Statistical analysis

Comparisons by HCA status for unmatched population used Fisher’s exact test for binary variables and Wilcoxon rank-sum or Mood’s median tests for continuous variables. Kaplan-Meier estimates were generated for 3-year post-discharge survival, with curves compared using the log-rank test. Continuous variables were expressed as a mean ± SD or median (IQR) and discrete data were presented as frequencies and percentage. Normality of continuous variables were assed based on Anderson-Darling test, histogram, and box plot.

Because the study spans 10 years, several variables contained missing data (75-24% for medical history, 4%-8% for malperfusion syndromes, 22%-27% for dissection extent, and 12%-30% for operative measures). Given incremental changes to IRAD data collection over 25 years, some fields were incompletely captured. Analyses used available-case denominators without assuming missing values. For propensity score matching, multiple imputation (100 datasets) was performed for missing medical history, presenting symptoms, and malperfusion data. This produced 100 matched cohorts; the number of matched pairs ranged from 94 to 101.

Baseline covariates were selected a priori for inclusion in the propensity score model based on clinical relevance and their potential influence on both operative strategy and perioperative outcomes. The matching cohort excluded HCA patients who underwent complete arch replacement or frozen elephant trunk (FET), as these cannot be performed with a closed distal anastomosis, as well as patients missing key matching variables (arterial cannulation site, aortic root replacement status, distal extent of dissection). For each of the 100 imputed datasets, propensity scores were estimated using multivariable logistic regression incorporating age, sex, BMI, Marfan syndrome, peripheral arterial disease, chronic renal insufficiency, 5 malperfusion syndromes, 4 arterial cannulation sites, any aortic root replacement, and 5 measures of distal dissection extent. Patients were matched 1:1 (HCA:no HCA) using greedy nearest-neighbour matching with a calliper of 0.1 times the standard deviation of the logit PS. Covariate balance was assessed using absolute standardized mean differences (ASMDs), with values <0.1 indicating adequate balance.

Matching was also performed using 4:1, 3:1, and 2:1 ratios with a calliper of 0.2, all of which produced comparable balance. Bivariate comparisons in the matched cohorts used paired *t*-tests for continuous variables and McNemar’s test for binary variables. Propensity matching was conducted separately across all 100 imputed datasets, and matched results were pooled using Rubin’s rules.^22^ Covariate balance was assessed by computing ASMDs within each imputed dataset and reporting the mean ASMD across all 100 datasets.

Kaplan-Meier survival curves for the matched cohorts were analysed using a log-rank test stratified by matched pairs. Survival probabilities were normalized, pooled across imputed datasets, and back-transformed for reporting. All analyses were performed in SAS 9.4 (SAS Institute, Cary, NC, United States), with statistical significance defined as *P* < .05.

Follow-up data were available for approximately 63% of the cohort. Among these patients, the clamp-on group had a longer median follow-up duration (35.7 vs 24.5 months).

## RESULTS

### Patient demographics and operative data

Of 2559 patients identified in the IRAD-IVC between 2010 and 2020, 312 were excluded due to missing circulatory arrest data, 244 due to prior cardiac surgery, and 28 due to both, yielding a final cohort of 2031 patients (**Figure 1**). Among these, 1900 (93.5%) underwent repair with HCA and an open distal anastomosis, whereas 131 (6.5%) underwent repair without removal of the aortic cross-clamp. Baseline characteristics are summarized in **Table 1**.

**Table 1.** Patient Demographics and Clinical Presentation

Variable	All patients			Propensity-matched patients		
	Clamp On (*n* = 131)	Clamp Off (*n* = 1900)	ASMD	Clamp On (*n* = 94-101)	Clamp Off (*n* = 94-101)	ASMD
Age, years, mean ± SD	57.2 ± 16.5	60.8 ± 14.0	0.23	56.9 ± 14.1	57.0 ± 14.6	0.07
Female gender	53 (40.5)	661 (34.8)	0.12	40.7	40.7	0.0
Body mass index, kg/m^2^	29.1 ± 7.7	29.0 ± 6.9	0.01	28.8 ± 7.3	29.0 ± 7.2	0.08
Hypertension	87 (71.3)	1322 (75.0)	0.08	68.1	73.0	0.12
Diabetes mellitus	11 (9.2)	198 (11.6)	0.08	9.3	13.1	0.12
Peripheral arterial disease	5 (4.3)	41 (2.5)	0.10	3.3	3.7	0.07
Current or former smoker	54 (50.9)	723 (50.5)	0.01	51.0	51.7	0.09
Chronic obstructive pulmonary disease	10 (8.5)	117 (7.0)	0.06	6.6	6.9	0.08
Chronic renal insufficiency	11 (9.2)	115 (6.9)	0.09	9.5	10.5	0.08
Bicuspid aortic valve	7 (5.9)	41 (2.4)	0.18	3.3	3.9	0.06
Marfan syndrome	5 (4.3)	34 (2.0)	0.13	4.0	3.7	0.07
Malperfusion syndrome	38 (29.0)	515 (27.8)	0.05	26.0	22.9	0.09
Stroke	3 (2.4)	95 (5.2)	0.15	3.6	4.1	0.08
Altered level of consciousness	13 (10.2)	168 (9.2)	0.04	8.7	9.6	0.08
Lower extremity ischaemia	17 (13.5)	285 (15.6)	0.06	11.8	11.4	0.08
Peripheral neuropathy/spinal cord ischaemia	11 (8.7)	150 (8.3)	0.01	9.0	8.8	0.08
Mesenteric ischaemia	6 (4.9)	45 (2.6)	0.12	3.2	3.2	0.06
Most proximal extent of dissection						
Aortic root	78 (68.4)	967 (66.0)	0.05	68.8	64.8	0.11
Sinotubular junction	14 (12.3)	141 (9.6)	0.09	12.1	8.1	0.15
Aorta proximal to innominate artery	20 (17.5)	325 (22.2)	0.12	17.1	25.8	0.21
Aortic arch	2 (1.8)	22 (1.5)	0.02	2.1	0.6	0.15
Most distal extent of dissection						
Aorta proximal to innominate artery	34 (31.8)	107 (7.7)	0.63	27.0	26.8	0.05
Aortic arch	19 (17.8)	231 (16.7)	0.03	18.3	18.0	0.07
Descending thoracic aorta	9 (8.4)	235 (17.0)	0.26	9.2	9.0	0.07
Abdominal aorta	28 (26.2)	414 (29.9)	0.08	28.3	29.5	0.07

Values expressed are *n* (%), % of patients, or median (IQR), unless otherwise specified.

Abbreviations: ASMD, absolute standardized mean difference; IQR, interquartile range.

Before matching, the clamp-on cohort was younger and had higher rates of female sex, bicuspid aortic valve, and preoperative stroke. Anatomical differences were also observed, with fewer clamp-on patients having a most proximal extent proximal to the innominate artery, more having this level as the most distal extent, and fewer demonstrating extension into the descending thoracic aorta. These imbalances were reflected by multiple covariates with ASMD values >0.1.

Because a small subset of patients had arch-limited proximal extent, a sensitivity analysis excluding these cases was performed and yielded no meaningful changes in mortality, stroke, or other major perioperative outcomes.

After matching, baseline variables were well balanced. All ASMDs were <0.1, indicating adequate covariate balance. Minor residual anatomical differences persisted in the distribution of proximal extent. The clamp-on cohort also maintained slightly lower rates of hypertension and diabetes, although these differences were modest (ASMD 0.12).

Intraoperative characteristics are summarized in **Table 2**. Before matching, the clamp-on cohort underwent repair at warmer temperatures and had shorter cardiopulmonary bypass and aortic cross-clamp times compared with the clamp-off group. As expected, no clamp-on patients required circulatory arrest, whereas all clamp-off patients did, most with antegrade cerebral perfusion. By definition, no clamp-on patients underwent hemiarch replacement, whereas all clamp-off patients included in the matched cohort underwent ascending aortic replacement with an open distal anastomosis (hemiarch). Patients undergoing total arch replacement or FET were excluded, as these procedures mandate circulatory arrest and an open distal anastomosis and are not compatible with a clamp-on strategy. Isolated ascending aortic replacement was comparable between the groups, whereas aortic root replacement with a composite valve-graft conduit was performed more frequently in the clamp-on cohort. When aortic valve replacement was performed, mechanical prostheses were used more often, and mitral valve procedures were also more common in the clamp-on group. Prior to matching, clamp-on patients were more likely to undergo femoral, innominate, or direct aortic cannulation and less likely to undergo axillary cannulation.

**Table 2.** Operative Data

Variable	All patients			Propensity-matched patients		
	Clamp On (*n* = 131)	Clamp Off (*n* = 1900)	ASMD	Clamp On (*n* = 94-101)	Clamp Off (*n* = 94-101)	ASMD
Cardiopulmonary bypass time, minutes	131.0 (110.0-176.0)	192.0 (152.0-240.0)	0.82	128.4 (110.4-171.2)	183.6 (144.4-231.6)	0.40
Aortic cross-clamp time, minutes	88.0 (69.0-127.0)	105.0 (71.0-144.0)	0.26	85.6 (64.6-114.1)	89.3 (63.2-126.8)	0.09
Circulatory arrest time, minutes	–	32.0 (22.0-45.0)	–	–	26.6 (18.0-36.9)	–
Lowest systemic temperature, °C	30.0 (27.0-32.2)	22.1 (18.0-26.0)	1.52	29.8 (26.7-31.9)	20.1 (17.9-25.0)	0.64
Cerebral perfusion during circulatory arrest	–	1606 (86%)	–	–	76.6	–
Antegrade	–	1091 (73.0)	–	–	75.6	–
Retrograde	–	403 (27.0)	–	–	24.4	–
Aortic procedure performed						
Isolated replacement of ascending aorta	52 (39.7)	757 (39.8)	0.002	31.2	37.1	0.13
Aortic root replacement with composite valve-graft conduit	31 (25.4)	347 (23.1)	0.05	27.1	22.6	0.12
Valve-sparing aortic root replacement	9 (7.1)	277 (16.9)	0.30	7.1	12.2	0.17
Aortic valve replacement	39 (31.2)	519 (31.6)	0.01	34.6	28.1	0.15
Mechanical	18 (46.2)	183 (36.9)	0.19	50.0	43.0	0.19
Bioprosthetic	21 (53.8)	307 (61.9)	0.16	50.0	57.0	0.19
Total arch replacement	–	311 (18.4)	–	–	–	–
Concomitant (non-aortic) procedures performed						
Coronary artery bypass grafting	12 (9.6)	209 (12.2)	0.08	8.2	9.6	0.08
Mitral valve repair or replacement	4 (3.2)	12 (0.7)	0.18	1.1	0.9	0.08
Arterial cannulation site						
Femoral	58 (46.0)	541 (32.5)	0.28	43.0	42.2	0.07
Axillary	29 (23.0)	742 (44.6)	0.47	27.4	28.0	0.07
Aortic	26 (20.6)	264 (15.9)	0.12	18.4	18.3	0.06
Innominate	12 (9.5)	99 (6.0)	0.13	10.1	10.6	0.07

Values expressed are *n* (%), % of patients, or median (IQR), unless otherwise specified.

In the matched cohort, all clamp-off patients underwent hemiarch replacement; no clamp-on patients did, as hemiarch requires cross-clamp removal by definition.

Abbreviations: ASMD, absolute standardized mean difference; IQR, interquartile range.

After propensity matching, cardiopulmonary bypass time remained shorter in the clamp-on cohort, whereas aortic cross-clamp times were comparable. Clamp-on repairs were performed at warmer temperatures. Isolated ascending aortic replacement was slightly more frequent in the clamp-off group, although this difference was modest (ASMD 0.13). Clamp-on patients more often underwent aortic root replacement with a composite valve-graft conduit and aortic valve replacement, whereas valve-sparing root replacement was more common in the clamp-off group.

### Clinical outcomes

Perioperative outcomes are presented in **Table 3**.

**Table 3.** Clinical Outcomes

Variable	All patients			Propensity-matched patients		
	Clamp On (*n* = 131)	Clamp Off (*n* = 1900)	*P-*value	Clamp On (*n* = 94-101)	Clamp Off (*n* = 94-101)	*P-*value
In-hospital mortality	9 (6.9)	257 (13.6)	.03	5.1	11.0	.22
Stroke	7 (5.8)	190 (11.4)	.07	5.8	10.2	.32
Bleeding requiring reoperation	11 (10.7)	130 (8.8)	.48	11.3	8.4	.55
Respiratory insufficiency	30 (28.8)	424 (28.1)	.91	26.2	27.1	.55
New renal failure requiring dialysis	2 (2.0)	108 (7.4)	.04	1.3	5.3	.37
Length of postoperative stay, days	9.0 (6.0-16.5)	11.0 (7.0-18.0)	.03	8.0 (5.9-14.5)	10.1 (6.5-16.3)	.11

Values expressed are *n* (%), % of patients, or median (IQR), unless otherwise specified.

Abbreviations: ASMD, absolute standardized mean difference; IQR, interquartile range.

For the overall cohort, in-hospital mortality was higher in the clamp-off group before matching. After propensity matching, in-hospital mortality was comparable between the groups. Rates of stroke and other major perioperative complications were likewise comparable in the matched cohorts.

**Figure 2.**
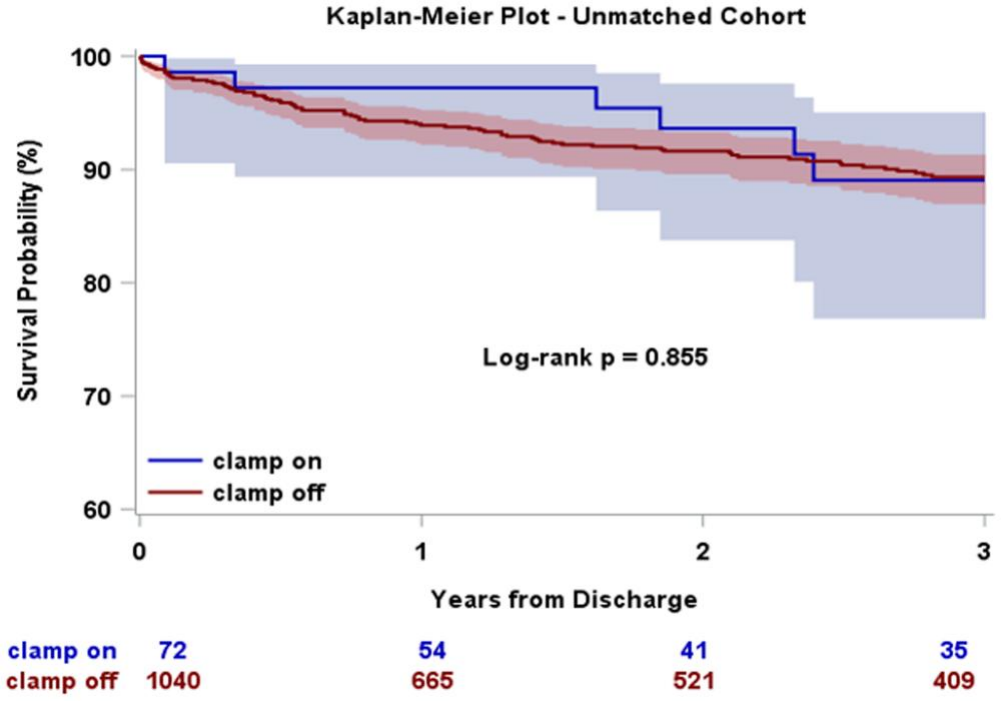
Three-Year Post-Discharge Survival for Unmatched Cohort, Clamp On vs Clamp Off During Ascending Aortic Replacement for ATAAD

**Figure 3.**
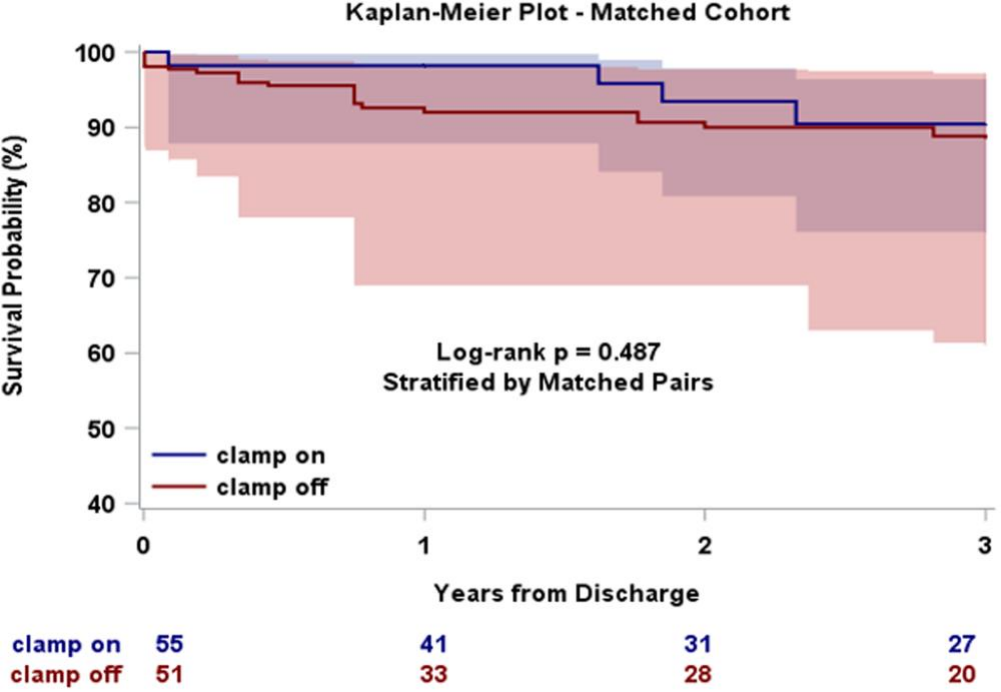
Three-Year Post-Discharge Survival for Matched Cohort, Clamp On vs Clamp Off During Ascending Aortic Replacement for ATAAD

**Figure 4.**
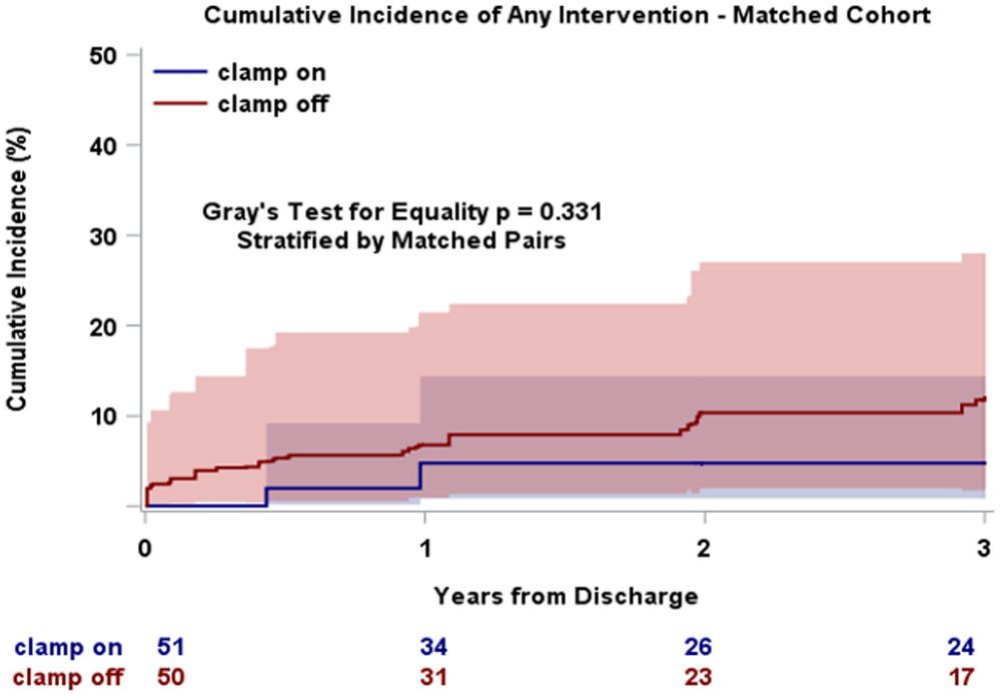
Cumulative Incidence of Aortic Reintervention Following Discharge in the Propensity-Matched Cohort (Clamp On vs Clamp Off). Reintervention includes any surgical or endovascular aortic procedure. Curves compared using Gray’s test stratified by matched pairs

Three-year post-discharge survival did not differ between the groups, both before and after matching (**Figures 2 and 3**). Discrete Kaplan-Meier survival estimates at 1, 2, and 3 years for both cohorts are provided in **Table S1**. The cumulative incidence of aortic reintervention following discharge (surgical or endovascular) was also similar between the groups in the unmatched (Gray’s test *P* = .28; **Figure S1**) and matched cohorts (Gray’s test *P* = .33; **Figure 4**).

## COMMENTS

This study indicates that a distal anastomosis performed using an open distal anastomotic technique (“Clamp Off”) under varying levels of HCA have comparable short-term and mid-term results when compared to maintaining the aortic cross-clamp on for the duration of the repair (“Clamp On”), consistent with findings by some studies,^17–21^ but not all.^16^

Examining the impact of surgical techniques on ATAAD is challenging due to numerous unpredictable factors that influence outcomes. Further, as Geirsson et al noted,^16^ there is a lack of contemporary and robust data on distal anastomotic techniques, with only 6 studies available in literature—5 reporting no statistically significant differences in operative morbidity or mortality.^17–21^ Although current surgical guidelines endorse an open distal anastomosis,^14^^,^^15^ the supporting literature is neither overwhelming nor entirely consistent. The 2 cited studies by our surgical guidelines include Geirsson et al’s NORCAAD analysis,^16^ which identified increased operative, 30-day, and in-hospital mortality with Clamp On operations in a higher-risk patient population, and Malvindi et al’s study,^17^ which found no significant difference in mortality. In fact, while not statistically significant, the intraoperative mortality (2% vs 1%; *P* = .90) and in-hospital mortality (14% vs 8%; *P* = .23) were higher in the clamp-off group.

Although the literature does not overwhelmingly support performing an open distal anastomosis, its widespread adoption is driven by several practical benefits. These include a bloodless field, better visualization for intraoperative decision-making regarding arch reconstruction, and the ability to extirpate the aortic cannulation and/or cross-clamp site, reducing the risk of later aneurysmal degeneration in compromised aortic tissue.

Registry data indicate that a proportion of ATAAD repairs in the United States are performed without circulatory arrest.^1^ Contemporary registry data suggest that this practice persists across institutions. When applied to carefully selected patients, a clamp-on strategy may yield acceptable outcomes, particularly given prior reports demonstrating comparable mortality and, in some series, lower morbidity.^16–21^ In patients who later require more extensive arch intervention, referral to specialized aortic centres remains appropriate.

While more extensive arch replacement may be appropriate in selected cases, operative strategy is influenced by patient anatomy, clinical presentation, and institutional practice patterns. In this international cohort, outcomes following a clamp-on strategy were not significantly different from those observed with an open distal anastomosis in carefully selected patients. These findings are consistent with several prior reports^17–21^ and suggest that, in selected cases, a clamp-on approach may represent an acceptable operative strategy.

At the same time, the potential impact of distal repair strategy on long-term aortic remodelling remains an important consideration. Total arch replacement may improve remodelling in ATAAD^23^^,^^24^; however, contemporary data from the STS Adult Cardiac Surgery Database,^25^ IRAD,^9^ and Canadian registries^26^ indicate that over 75% of cases are repaired with a hemiarch technique. Distal anastomotic new entry (DANE) tears have been reported in 40%-70% of these repairs^27^^,^^28^ and are associated with poor remodelling and accelerated growth.^27–30^ Accordingly, our findings should not be interpreted as endorsing untreated arch pathology; rather, they reflect contemporary practice patterns and suggest that, in carefully selected patients, a clamp-on strategy can achieve comparable short- and mid-term outcomes. The long-term aortic implications of distal repair strategy were not evaluated in this analysis and warrant further investigation.

This study has several limitations. Although multicentre and international, the analysis is retrospective and observational. Not all aortic centres contribute to IRAD, data are not externally audited, and many patients were excluded because of missing critical variables. Additionally, 22%-27% of data on dissection extent were missing. Although multiple imputation was performed prior to propensity matching, image-derived and operative variables were not imputed, and patients with missing data for these variables were excluded from the matching model. Accordingly, residual confounding related to dissection extent cannot be fully excluded. Furthermore, the study was not powered to detect small differences in stroke or other infrequent outcomes. Some included cases also had incomplete information, particularly regarding adjunctive cerebral perfusion during HCA. Perfusion and cerebral protection strategies varied widely in the open distal group but were uniform in the clamp-on group, introducing heterogeneity. Given the lack of consensus on optimal cerebral perfusion during HCA, these differences should be considered when interpreting comparisons between the groups.

In addition, follow-up completeness was limited, with post-discharge survival data available for approximately 63% of patients. Survival outcomes were not imputed, and loss to follow-up may introduce attrition bias and reduce power to detect differences in mid-term outcomes. These findings should therefore be interpreted with caution.

In this international registry analysis, ascending aortic replacement for ATAAD performed without circulatory arrest was associated with comparable early and mid-term outcomes in a highly selected patient population not undergoing arch intervention. These findings do not establish superiority of one distal anastomotic strategy over another, but suggest that, in carefully selected cases, a clamp-on approach may represent a reasonable operative option. Further prospective and adequately powered studies are needed to better define patient selection and long-term outcomes.

## Supplementary Material

ivag093_Supplementary_Data

## Data Availability

Data available on request. The data underlying this article will be shared on reasonable request to the corresponding author.

## ABBREVIATIONS

ATAAD: Acute type A acute aortic dissection
BMI: Body mass index
CPB: Cardiopulmonary bypass
EACTS: European Association for Cardio-Thoracic Surgery
HCA: Hypothermic circulatory arrest
IQR: Interquartile range
IRAD: International Registry of Acute Aortic Dissection
IVC: Interventional cohort
PS: Propensity score
RCP: Retrograde cerebral perfusion
STS: Society of Thoracic Surgeons
