# Supplementary material for: Clamp On vs Off Impact of Distal Anastomotic Technique During Ascending Aortic Replacement in Acute Type A Aortic Dissection: IRAD Insights
Source: Interdiscip Cardiovasc Thorac Surg. 2026 Apr 3;41(4):ivag093. doi: 10.1093/icvts/ivag093 (PMC13099637; doi:10.1093/icvts/ivag093)
Supplement: ivag093_Supplementary_Data [file ivag093_supplementary_data.docx]

**Supplementary Table S1.** Kaplan–Meier survival probabilities at 1, 2, and 3 years for both clamp-on and clamp-off cohorts.

| **Year** | **Clamp-Off Survival (%)** | **95% CI** | **SE** | **Clamp-On Survival (%)** | **95% CI** | **SE** |
| --- | --- | --- | --- | --- | --- | --- |
| 1 | 93.9 | 92.2–95.2 | 0.008 | 97.2 | 89.3–99.3 | 0.019 |
| 2 | 91.6 | 89.6–93.3 | 0.009 | 93.6 | 83.7–97.6 | 0.031 |
| 3 | 89.3 | 86.9–91.3 | 0.011 | 89.1 | 76.8–95.0 | 0.043 |

| **Year** | **Clamp-Off Survival (%)** | **95% CI** | **SE** | **Clamp-On Survival (%)** | **95% CI** | **SE** |
| --- | --- | --- | --- | --- | --- | --- |
| 1 | 94.7 | 72.2–99.1 | 0.047 | 98.2 | 87.9–99.7 | 0.018 |
| 2 | 93.9 | 68.1–99.0 | 0.055 | 93.5 | 80.9–97.9 | 0.037 |
| 3 | 89.5 | 65.1–97.2 | 0.069 | 90.5 | 76.1–96.4 | 0.046 |

**Supplementary Table Legends**

**Supplemental Table S1:** Kaplan–Meier survival estimates at 1, 2, and 3 years for clamp-off (open distal with hypothermic circulatory arrest) and clamp-on cohorts in both unmatched and propensity-matched populations. Survival probabilities are presented with corresponding 95% confidence intervals (CI) and standard errors (SE).

**Supplementary Figures**

Supplementary Figure S1


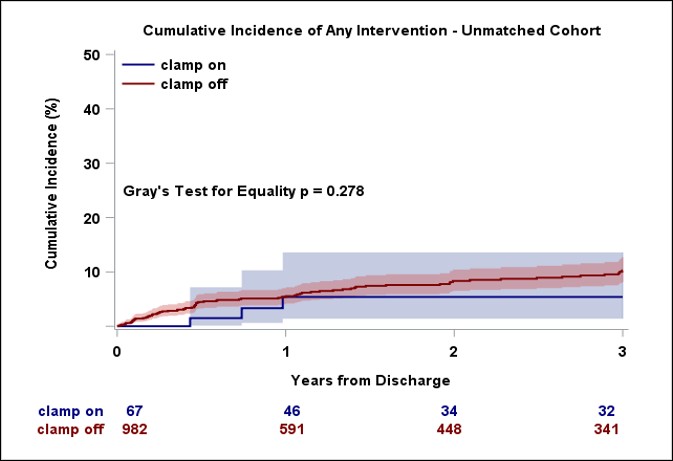


**Supplementary Figure Legend S1:** Cumulative incidence of aortic reintervention following discharge in the unmatched cohort (Clamp On vs Clamp Off). Reintervention includes any surgical or endovascular aortic procedure. Curves compared using Gray’s test stratified by matched pairs.
